# Unlocking the potential of CD70 as a novel immunotherapeutic target for non-small cell lung cancer

**DOI:** 10.18632/oncotarget.3880

**Published:** 2015-04-19

**Authors:** Julie Jacobs, Karen Zwaenepoel, Christian Rolfo, Jolien Van den Bossche, Christophe Deben, Karen Silence, Christophe Hermans, Evelien Smits, Paul Van Schil, Filip Lardon, Vanessa Deschoolmeester, Patrick Pauwels

**Affiliations:** ^1^ Center for Oncological Research Antwerp, Center for Oncological Research Antwerp (CORE), Antwerp University, Wilrijk, Belgium; ^2^ Department of Pathology, Antwerp University Hospital, Edegem, Belgium; ^3^ Department of Oncology, Antwerp University Hospital, Edegem, Belgium; ^4^ Phase 1-Early Clinical Trials Unit, Antwerp University Hospital, Edegem, Belgium; ^5^ arGEN-X BVBA, Ghent, Belgium; ^6^ Laboratory of Experimental Hematology (LEH), Vaccine and Infectious Disease Institute, Antwerp University, Wilrijk, Belgium; ^7^ Department of Thoracic and Vascular Surgery, Antwerp University Hospital, Edegem, Belgium

**Keywords:** CD70, NSCLC, immunotherapy, targeted therapies

## Abstract

Although normally restricted to activated T and B cells and mature dendritic cells, constitutive expression of CD70, a member of the tumor necrosis family, has been described in both hematological and solid tumors, where it increases tumor cell and regulatory T cell survival by signaling through its receptor, CD27.

We have assessed the co-expression of CD70 and CD27 in non-small cell lung cancer (NSCLC) by immunohistochemistry to explore a correlation between expression of the protein and tumor histologic subtype, genetic aberrations and prognosis. Furthermore, we tested the ability of ARGX-110, a CD70-blocking antibody, to induce NK cell-mediated cytotoxicity.

Our results revealed CD70 expression on the surface of both primary and metastatic NSCLC tumor cells and in the tumor microenvironment. Moreover, CD27-expressing tumor infiltrating lymphocytes were found adjacent to the tumor cells, suggesting active CD70-mediated signaling. Finally, we have shown that ARGX-110, has potent cytotoxic effects on CD70^+^ NSCLC cell lines.

## INTRODUCTION

Over the last decades, a revolution in the perspective of lung cancer treatment has taken place. Improvements in molecular profiling techniques has led to the identification of distinct molecular subtypes of non-small cell lung cancer (NSCLC), for which biology-driven targeted therapies can be harnessed. For example, orally administered targeted agents directed against the tyrosine kinase domain of the epidermal growth factor receptor (EGFR) have resulted in improved progression-free survival as opposed to best supportive care in advanced lung cancer [1-3]. Furthermore, Shaw et al. [4] showed marked antitumor activity targeting anaplastic lymphoma kinase (ALK) rearrangements in NSCLC. Despite these novel therapies, clinical anti-tumor responses remain of limited duration and are only applicable to a minority of patients, harboring targetable oncogenic mutations in the tumor cells [5]. As a result NSCLC, accounting for an estimated 85% of lung cancers, retains its position as the most lethal type of cancer worldwide with marginally improving 5-year overall survival rates for newly diagnosed cases, remaining below 20% [6, 7]. Hence, alternative strategies to treatment of NSCLC and improvements of patient survival are in high demand and therefore, rationally designed immunotherapeutic strategies are being explored.

Recently, much interest has been generated by the clinical results associated with inhibition of immune checkpoint proteins by antibodies directed against cytotoxic T lymphocyte antigen-4 (CTLA-4) and programmed death (ligand) -1 (PD-1/PDL-1) [8, 9]. This study will focus on the CD70-CD27 signaling pathway, as an interesting new target to enhance anti-tumoral immune responses in NSCLC.

Under normal physiological conditions, CD27, a member of the tumor necrosis factor receptor (TNFR) superfamily, plays a co-stimulatory role in promoting T cell expansion and differentiation through activation of the NFκB pathway [10, 11]. Consequently, expression of its ligand, CD70, is tightly regulated and only transiently expressed on activated T cells, B cells and mature dendritic cells [12]. In contrast to a total lack of constitutive CD70 expression in normal tissue, CD70 overexpression has been documented in diverse tumor types such as renal cell carcinoma [13], glioblastoma [14], and hematological malignancies [11]. In the latter, CD70 overexpression has even been implicated in tumor cell proliferation and survival mediated through its interaction with CD27 [15, 16]. Moreover, Claus et al. [17] demonstrated evasion of immune surveillance by recruitment of CD27^+^ regulatory T cells (Treg) to the tumor site. Consequently, this surface factor might be an interesting and specific therapeutic target in addition to its role as prognostic biomarker. Furthermore, upon binding of CD70 to CD27, soluble CD27 (sCD27) is cleaved off by metalloproteinases and has been detected in serum, plasma, and urine samples from healthy individuals, and at increased levels in patients with autoimmune diseases [17, 18]. In fact, increased levels of sCD27 have been reported to correlate with poor prognosis in various hematological malignancies [16, 19]. Therefore, this study was designed to evaluate the potential use of sCD27 as a diagnostic biomarker for prognosis in solid malignancies.

Monoclonal antibodies (mAb), able to block immune checkpoint proteins, have been associated with objective clinical responses against various types of cancer and hold great promise as novel cancer therapeutics [20]. The constitutive overexpression of CD70 on tumor cells and its absence on normal tissue, has led to the development of two different anti-CD70 monoclonal antibodies (mAb), SGN-CD70A and ARGX-110. ARGX-110, a blocking IgG1 mAb which is currently in a Phase 1b clinical trial, has been developed with enhanced antibody-dependent cellular cytotoxicity (ADCC), endowing the antibody with the ability to deplete CD70 expressing tumor cells as a mode of action additional to its ability to inhibit immune checkpoint function [21, 22].

To our knowledge the immunotherapeutic potential of CD70 in NSCLC has never been studied before. In this study, we report the expression of CD70 on malignant cells of lung neoplasms and the therapeutic benefit of ARGX-110 in CD70^+^ NSCLC cell lines. In addition, we have demonstrated the expression of the CD70 receptor, CD27, in the microenvironment of the tumor and the presence of soluble CD27 in NSCLC patient sera.

## RESULTS

### CD70 protein expression in primary NSCLC

**Figure 1:**
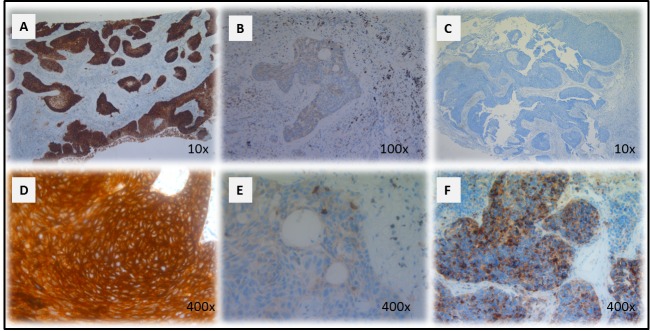
CD70 staining in primary NSCLC Representative sections of a NSCLC tumour classified as strong **A.** and weak **B.** for CD70 expression based on dye intensity. A CD70 negative NSCLC sample is shown in **C.** Membranous **D.** and cytoplasmic **E.** accentuation of CD70 expression in NSCLC specimens. CD70 expression in a neuro-endocrine NSCLC primary biopsy (F). Magnitude is depicted in the lower right corner.

A total of 49 surgically resected lung cancer specimens were analyzed by immunohistochemistry (IHC) for CD70 expression. Overall, 8 (16.3%) samples scored positive for CD70 (Table 2). IHC analysis showed specific anti-CD70 mAb binding to NSCLC cells, while adjacent normal lung tissue did not show CD70 staining, demonstrating the absence of CD70 expression in non-malignant cells. Furthermore, varying distribution patterns of CD70 expression were found in NSCLC cells including cytoplasmic (55% of cases) and membranous expression (45% of cases) with differences in intensity. Representative examples of the different types of CD70 expression are shown in Figure 1A-1E).

**Table 1:** Patient characteristics. Overview of the clinico-pathological characteristics of patients (1^st^ column), biopsies (2^nd^ column) and serum samples (3^th^ column)

	Patients n=53(%)	Biopsies n=65 (%)	Serum n=19 (%)
**Gender**			
Male	34 (64%)	40 (62%)	16 (84%)
Female	19 (36%)	25 (38%)	3 (16%)
**Histologic Type**			
Adenocarcinoma	36 (68%)	45 (70%)	10 (53%)
Squamous	15 (28%)	18 (28%)	8 (42%)
Large cell	1 (2%)	1 (1%)	1 (5%)
Neuro-endocrine	1 (2%)	1 (1%)	0 (5%)
**Differentiation**			
Weak	13 (25%)	14 (22%)	7 (37%)
Moderate	21 (40%)	24 (37%)	8 (42%)
Strong	9 (17%)	10 (15%)	1 (5%)
**TxNxMx**			
T1	10 (19%)	11 (17%)	3 (16%)
T2	18 (34%)	19 (29%)	8 (42%)
T3	15 (28%)	15 (23%)	7 (37%)
T4	5 (9%)	5 (8%)	1 (5%)
**Relapse**			
Lung	4 (8%)	2 (3%)	0 (0%)
**Metastasis**			
Lymph node	20 (34%)	8 (12%)	6 (32%)
Organ	8 (15%)	2 (3%)	0 (0%)
**Genetic aberrations**			
EGFR mutation	12 (23%)	15 (23%)	2 (11%)
Alk translocation	3 (6%)	3 (5%)	0 (0%)

**Table 2:** Relation between sCD27 levels or CD70 expression and clinico-pathological features Data about CD70 expression was available for 49 patients, except for information about tumour-infiltrating lymphocytes which was only available for 42 patients

	sCD27 levels			CD70 expression		
	Positive	Negative	p-value	Positive	Negative	p-value
**Gender**						
Male	3 (20%)	12 (80%)	1.000	6 (18%)	28 (82%)	1.000
Female	1 (25%)	3 (75%)		2 (13%)	13 (87%)	
**Smoking habit**						
No smoker	1 (33%)	2 (67%)	0.576	3 (27%)	8 (73%)	0.536
Smoker	3 (23%)	10 (77%)		4 (13%)	26 (87%)	
Unknown	0 (0%)	3 (100%)		1 (13%)	7 (87%)	
**Histologic Type**						
Adenocarcinoma	2 (20%)	8 (80%)	1.000	3 (9%)	29 (91%)	0.054
Squamous	2 (22%)	7 (78%)		4 (27%)	11 (73%)	
Large cell	NA	NA		0 (0%)	1 (100%)	
Neuro-endocrine	NA	NA		1 (100%)	0 (0%)	
**Differentiation**			
Weak	2 (40%)	5 (60%)	0.094	3 (23%)	10 (77%)	0.507
Moderate	0 (0%)	8 (100%)		4 (19%)	17 (81%)	
Strong	0 (0%)	1 (100%)		1 (11%)	8 (89%)	
Unknown	2 (33%)	1 (67%)		0 (0%)	6 (100%)	
**TxNxMx**						
T1	0 (0%)	2 (100%)	0.790	3 (30%)	7 (70%)	0.096
T2	2 (22%)	7 (78%)		3 (16%)	16 (84%)	
T3	2 (29%)	5 (71%)		0 (0%)	15 (100%)	
T4	0 (0%)	1 (100%)		2 (40%)	3 (60%)	
**Lymph node**						
N0	2 (15%)	11 (85%)	0.090	6 (20%)	24 (80%)	0.645
N1	2 (67%)	1 (33%)		2 (17%)	10 (83%)	
N2	0 (0%)	3 (100%)		0 (0%)	5 (100%)	
Unknown	NA	NA		0 (0%)	2 (100%)	
**Stage**			
I	0 (0%)	6 (100%)	0.255	4 (29%)	10 (71%)	0.202
II-IV	4 (31%)	9 (69%)		4 (11%)	31 (89%)	
**TIL**						
<10%	0 (0%)	6 (100%)	0.622	1 (13%)	7 (87%)	0.557
10-50%	0 (0%)	1 (100%)		5 (18%)	23 (82%)	
>50%	4 (25%)	12 (75%)		0 (0%)	6 (100%)	
**CD70^+^ TIL**						
<10%	3 (60%)	2 (40%)	**0.044**	3 (12%)	23 (88%)	0.671
10-50%	1 (8%)	12 (92%)		3 (20%)	12 (80%)	
>50%	0 (0%)	1 (100%)		0 (0%)	1 (100%)	
**CD27^+^ TIL**						
<10%	2 (2%)	0 (0%)	**0.012**	0 (0%)	4 (100%)	0.499
10-50%	2 (15%)	11 (85%)		0 (0%)	4 (100%)	
>50%	0 (0%)	4 (100%)		6 (18%)	28 (82%)	

The potential relationship between CD70 expression and histological subtype or disease status was analyzed. From the 8 patients with CD70^+^ tumor cells, positivity correlated more frequently with squamous NSCLC (26.7% CD70^+^) in comparison to adenocarcinoma (9.4% CD70^+^). Remarkably, a large cell neuro-endocrine carcinoma biopsy was also found to be CD70 positive (Figure 1F), while a NSCLC not otherwise specified (NOS) biopsy showed no detectable CD70 expression. In addition, IHC analysis suggested preferential expression of CD70 in T4 stage lung cancer (2 out of 5 T4NxMx biopsies (40%) with >10% CD70 positivity). Moreover, 75% of cases with CD70^+^ tumor cells showed poor response to first-line treatment with a progression-free survival interval of less than 1 year (data not shown). Although a trend could be seen in the samples analyzed, no statistically significant correlation could be found between CD70 expression and clinicopathological factors. An overview is shown in Table 2.

In addition, for two patients, biopsies taken before and after administration of platinum-based chemotherapy, were analyzed for CD70 expression to determine whether the addition of chemotherapy could influence the expression pattern of this molecular marker. Results shown in Table 3 reveal that in more than 50% of tumor cells post-chemotherapy, weak CD70 expression was induced in comparison to pre-treatment samples.

**Table 3:** Influence of chemotherapy on CD70 expressing tumour cells in NSCLC

	Pre-biopsy			Post-biopsy		
N°	Intensity	% CD70^+^ TC	Distribution	Intensity	% CD70^+^ TC	Distribution
1	NS	0%	-	+	60%	C
2	NS	0%	-	+	80%	C

*Abbreviations: NS, no staining; +, weak staining intensity; C, cytoplasmic accentuation; TC, tumour cells

### CD70 protein expression and genetic aberrations

Of the 53 cases analyzed for *EGFR* mutations, 10 activating EGFR mutations (19del, L858R), 2 resistance EGFR mutations (20ins) and one acquired resistance mutation (T790M) were found. In addition, three biopsies with an ALK translocation were included in the study. Although no expression of CD70 was found in biopsies showing ALK translocations and resistance or activating EGFR mutations, the biopsy with a T790M mutation revealed CD70 positivity in the tumor cells. Results are shown in Table 4.

**Table 4:** Correlation of CD70 expressing tumour cells with genetic rearrangements in NSCLC

	CD70 expression	
	Positive	Negative
Alk translocation	0 (0%)	3 (100%)
EGFR mutation		
20INS	0 (0%)	2 (100%)
19DEL	0 (0%)	5 (100%)
L858R	0 (0%)	5 (100%)
T790M	1 (100%)	0 (100%)
Wild type	8 (22%)	29 (78%)
TOTAL	9/53	44/53

### Stability of CD70 protein expression during disease progression

**Figure 2:**
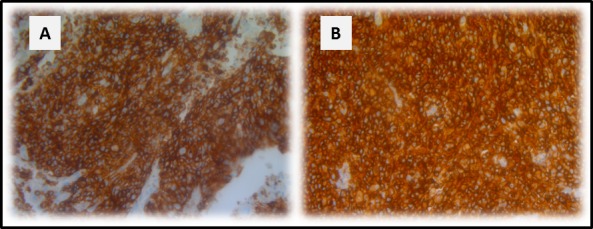
Stable CD70 protein expression in paired primary and metastatic NSCLC tissue CD70 protein expression in primary NSCLC tissue **A.** and small intestine metastatic tissue **B.** of the same patient. Magnitude x200.

For 10 primary tumor samples, matched tissue from metastatic sites was available, including 8 lymph nodes (LN) taken at time of primary tumor resection, 1 small intestine and 1 pleural fluid biopsy. The results, shown in Table 5, demonstrate comparable CD70 expression patterns between primary and metastatic tissues in 80% of cases. In a primary lung adenocarcinoma and a small intestine metastasis from the same patient, CD70 staining of equivalent intensity with clear membranous accentuation in more than 80% of the tumor cells was observed (Figure 2). In contrast, only 6 of 8 LN biopsies showed corresponding expression patterns of CD70 to their primary tumors

**Table 5:** CD70 protein expression levels in paired primary and metastatic NSCLC tissue

	Primary			Metastasis			
N°	Intensity	% CD70^+^ TC	Distribution	Tissue	Intensity	% CD70^+^ TC	Distribution
1	+++	90%	M	Intestine	+++	100%	M
2	++	80%	M	LN	NS	0%	-
3	NS	0%	-	LN	+	80%	C
4	NS	0%	-	LN	NS	0%	-
5	NS	0%	-	LN	NS	0%	-
6	NS	0%	-	LN	NS	0%	-
7	NS	0%	-	LN	NS	0%	-
8	NS	0%	-	LN	NS	0%	-
9	NS	0%	-	Pleaura	NS	0%	-
10	NS	0%	-	LN	NS	0%	-

*Abbreviations: NS, no staining; +, weak staining intensity; ++, moderate staining intensity; +++, strong staining intensity; M, membranous accentuation; C, cytoplasmic accentuation

### CD70/CD27 expression and tumor infiltrating lymphocytes (TILs)

**Figure 3:**
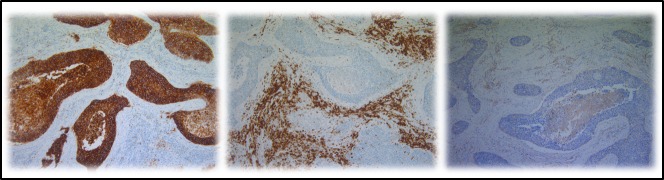
Representative IHC on serial cuts showing the expression of CD70, CD27 and FOXP3 From left to right: CD70 expression in the tumour cells of a primary NSCLC section, CD27 expression and FOXP3 expression in the tumour micro-environment. Magnification: 100x.

CD70 and CD27 expression in the tumor microenvironment was analyzed in 42 primary NSCLC samples. Overall, 55% of samples (23 of 42) were found to contain more than 10% CD70^+^ TILs in the tumor microenvironment. Subsequently, we assessed the expression of CD27 in these primary NSCLC specimens. Although, CD27^+^ tumor cells could not be found, over 90% of specimens (38 of 42) showed CD27 expression on TILs adjacent to the tumor cells. However the presence of CD27^+^ TILs did not appear to be associated with CD70 expression in the tumor cells (Figure 3).

Subsequently, the phenotype of CD27^+^ TILs was investigated in serial cuts of CD70^+^ and CD70^−^ tumor samples. A trend towards increased FOXP3^+^ lymphocyte infiltration was seen in CD70^+^ versus CD70^−^ samples (Figure 3). In addition, expression of CD4 and CD8 lymphocytes was studied in a subset of 5 patients including CD70^+^ and CD70^−^ biopsies, demonstrating an increased CD4/CD8 ratio in biopsies containing CD70^+^ tumor cells (data not shown).

### Serum sCD27 levels and overall survival (OS)

**Figure 4:**
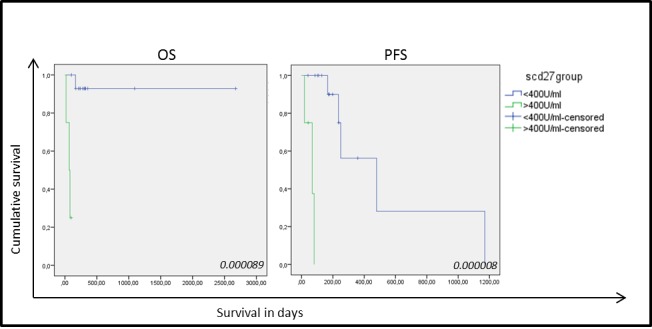
Kaplan-Meier curves for overall survival and progression-free survival based on sCD27 levels in 19 NSCLC samples Correlation of sCD27 levels with overall survival (OS) (left) and progression free survival (PFS) (right) in patients with high (green) and low (blue) sCD27 levels. *P*-values are giving in the right corner.

Nineteen serum samples were analyzed showing a mean sCD27 level of 358±257 U/ml (mean±SD) (range 174-1331 U/ml) with a median of 263 U/ml. The cut-off value for sCD27 was calculated as 407 U/ml by ROC analysis (sensitivity, 0.75; specificity, 0.067). Clinical follow-up data and clinicopathological characteristics were available for all NSCLC patients. No correlation was found between high sCD27 levels and tumor histology, differentiation, lymph node invasion or tumor staging, and although no association was found with patient gender or history of smoking, a significant correlation was found with increasing age (*P*-value: 0.033). Univariate analysis of sCD27 levels pre-treatment and relation with disease prognosis revealed a significantly shorter OS and progression-free survival (PFS) in patients with high sCD27 levels (*P*-value < 0.00001 and *P*-value: 0.024, respectively) (Figure 4). Furthermore, high serum sCD27 levels were significantly associated with low CD27 expression in TILs (*P*-value: 0.012) and low levels of CD70^+^ TILs (*P*-value: 0.044). No relation between sCD27 levels and CD70 expression on tumor cells could be detected (Table 2). Patients with high sCD27 levels and CD70^+^ tumor cells did show a significantly shorter OS (*P*-value: 0.000002) and PFS (*P*-value: 0.002) compared to patients with high sCD27 levels alone (data not shown).

### Analysis of CD70 expression by different methods

**Figure 5:**
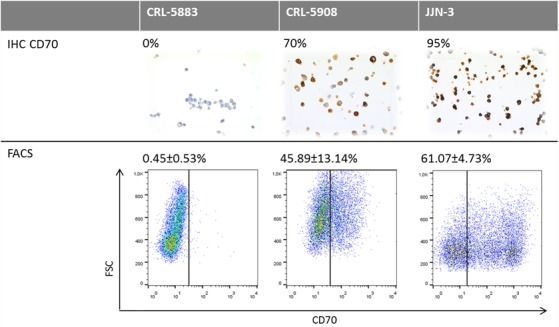
Comparative analysis of CD70 protein expression measured by flow cytometry and IHC Upper row: Representative sections of CD70 IHC on CRL-5883, CRL-5908 and JJN-3 cell lines. Magnification: 100x. Below: Dot plots showing expression of CD70 on CRL-5583, CRL-5908 and JJN-3 cell lines, using ARGX-110 coupled to anti-human IgG1 PE antibody. Debris was excluded from the analysis based on forward (FSC) and side scatter (SSC).

To investigate whether CD70 expression detected by IHC would serve as a reliable biomarker in the treatment of patients with the therapeutic antibody ARGX-110, IHC-based CD70 expression was compared to flow cytometric analysis using fluorescently-labelled ARGX-110. Two lung cancer cell lines (CRL-5883 and CRL-5908) as well as one positive control cell line (JJN3) were tested and the results, shown in Figure 5, show similar percentages of CD70 expression with both methods, indicating that IHC detection of CD70 expression is representative for binding of ARGX-110 to CD70.

### Anti-CD70 therapy for NSCLC *in vitro*

**Figure 6:**
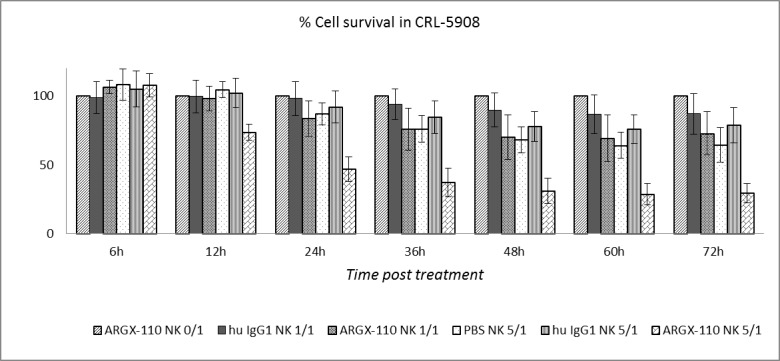
ADCC-induced cytotoxicity of ARGX-110 in CRL-5908 Mean percentage of cell lysis up to 72h after treatment as compared to ARGX-110 deprived of NK-cells, set at 100% in five different conditions. From left to right: ARGX-110; Isotype control + NK 1/1; ARGX-110 + NK 1/1; NK 5/1; Isotype control + NK 5/1; ARGX-110 + NK 5/1. In each test, two replicates of the same condition were used and run in parallel with NK cells from three different donors. Graph represents mean±SD.

**Figure 7:**
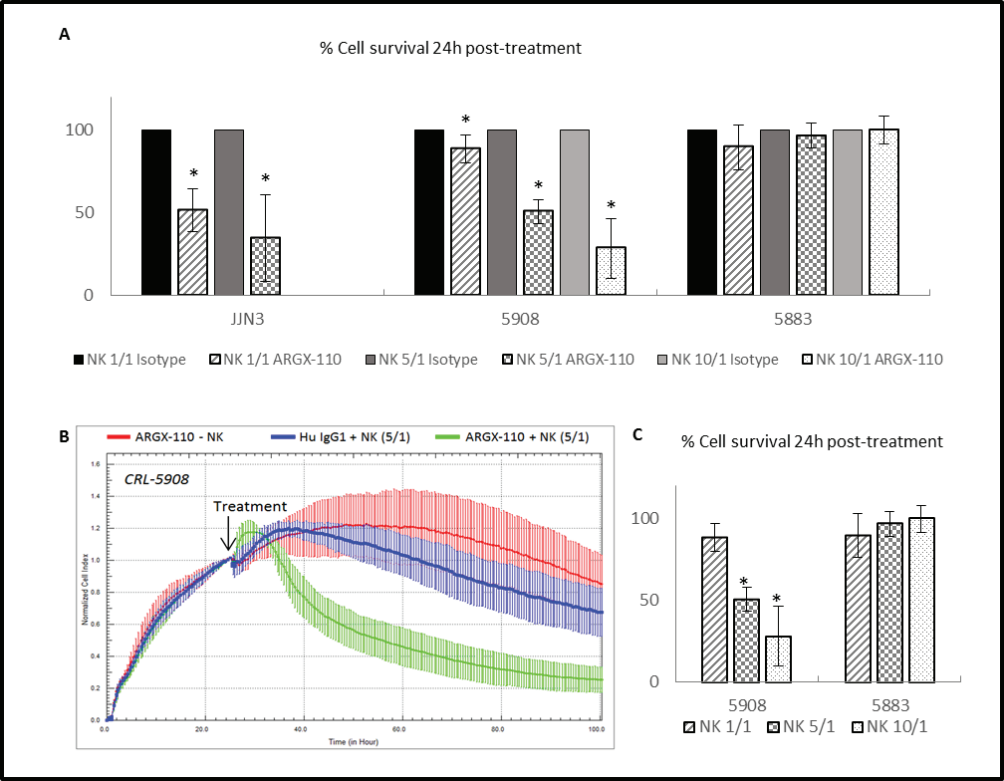
ADCC-induced cytotoxicity of ARGX-110 **A.** Percentage of cell survival 24h after treatment compared to the isotype control (set at 100%). Bars represent the mean±SD (*n* = 3). *p < 0.05: significant decrease in cell survival by ARGX-110 in comparison to isotype control with identical E/T ratio. **B.** CRL-5908 cells were incubated with ARGX-110 (red), NK cells (5/1) in combination with ARGX-110 (Green) or with isotype control (blue). The well impedance, expressed by the cell index as a measure of viability, was analysed using the xCELLigence system as described in the material and methods section. Cell indexes were normalized with the last point before compound addition, as indicated by the arrow. Graph represents mean±SD. **C.** Percentage of cell survival of a CD70+ (CRL-5908) and a CD70- (CRL-5883) cell line 24h after treatment with ARGX-110 with different ratios of NK cells (1/1 – 5/1 – 10/1). *P < 0.05: significant decrease in cell survival in comparison to E/T ratio of 1/1. For all experiments, two replicates of the same condition were measured and run in parallel with NK cells from three different donors.

The ADCC potential of ARGX-110, mediated by binding and activation of NK cells via mAb binding to FcγRIIIa, was evaluated *in vitro*. Peripheral blood NK cells, obtained from healthy volunteers, were spiked into cell lines (CRL-5908; CRL-5883; JJN-3) and monitored using the xCELLigence system in the presence or absence of ARGX-110 or an isotype control. Cell lines with high levels of CD70 expression (CRL-5908, JJN-3) showed a significant higher degree of ADCC-based killing by ARGX-110, in comparison to the isotype control. In contrast, ADCC effects of ARGX-110 on CRL-5883 cells expressing very low levels of CD70 were not observed. Figures 6 & 7 show that addition of NK cells alone had only minimal effects on lysis of CRL-5908 cells, but adding ARGX-110 resulted in an increase of 40% in cell lysis 24h post-treatment, which was maintained up to 72h post-treatment.

The difference in observed cell lysis between ARGX-110 and the isotype control was examined 24h post-treatment. Incubation at an effector (NK) to target (cell line) (E/T) ratio of 5/1 induced significantly greater cell lysis in the JJN-3 (34.69%) and CRL-5908 (50.66%) cell lines compared to the isotype control (Figure 7A and 7B). In addition, a significant increase in cytotoxicity mediated by ARGX-110 was detected using a higher E/T ratio (1/1 – 5/1 – 10/1) (Figure 7C).

## DISCUSSION

The clinical potential of immunotherapy in treating NSCLC patients has been recently demonstrated by mAbs targeting CTLA-4, PD-1 and PDL-1 [9]. Successful cancer immunotherapy requires tumor-specific proteins to elicit strong anti-tumor immune responses without inducing autoimmunity [23]. In this study, we are the first to reveal the immunotherapeutic potential of CD70 in the treatment of NSCLC. We have used IHC to demonstrate CD70 expression in the two most common histological NSCLC subtypes and shown consistent expression of the protein in 80% of metastatic tissue samples. Interestingly, no association was found between CD70 expression in NSCLC tumor samples and the presence of targetable gene arrangements, pointing towards a new subset of patients eligible for alternative therapy. In addition, we have shown the presence of its receptor, CD27, on TILs in the microenvironment of the tumor. Finally, we have shown *in vitro* that ARGX-110, a CD70-specific mAb with enhanced ADCC effects, is able to successfully mediate lysis of CD70^+^ cancer cells.

The absence of CD70 expression from normal lung tissue and its near absence from circulating lymphocytes, combined with its presence in 16% of NSCLC patient biopsies suggest a significant therapeutic window for CD70 targeted therapy. Moreover, we have demonstrated particular CD70 expression in stage T4 NSCLC (40% of cases) as well as in squamous cell carcinoma (27% of cases). Furthermore, we did not find evidence of concomitant expression of CD70 in biopsies carrying ALK translocations or activating EGFR mutations. Hence, the subset of patients whose tumors show CD70 positivity lack specific treatment options, necessitating the discovery of novel, rational targeted therapies. Based on these results CD70 may be a promising new target for therapy and thus merits further study.

CD70 expression was assessed in tumor biopsies taken both before and after platinum-based chemotherapy and shown to be clearly upregulated after treatment. It is an interesting hypothesis that chemotherapy could stimulate the expression of CD70 in malignant cells. If so, sequential combination regimens of conventional NSCLC treatment with anti-CD70 mAb therapy might induce a synergistic therapeutic effect in NSCLC. Although the mechanism through which chemotherapy induces CD70 expression is still largely unknown, our findings are similar to recent studies in ovarian cancer, where increased CD70 expression has been shown to associate with clinical resistance to cisplatin-based chemotherapy [24, 25].

Furthermore, in this report the expression of CD27, the receptor for CD70, was examined in tumor cells and their microenvironment. In normal physiological conditions, expression of CD27 is associated with naïve T and B cells and some memory T cells. However, more recently lung cancer studies have shown that CD27 expression on human CD4^+^CD25^+^ Tregs positively correlates with their suppressive activity *in vitro* and the expression of FOXP3 [26]. Moreover, Claus et al. [27] demonstrated that CD27-CD70 interactions increase the frequency of Tregs in the tumor microenvironment, reduce tumor-specific T cell responses and promote tumor cell growth when CD70 is expressed on TILs. In this study, CD70 expression was detected both in NCSLC cells (16%) and on TILs in the tumor microenvironment (55%). As a result, suppression of the anti-tumor response might easily be established through binding of CD70^+^ cells to CD27^+^ Tregs, leading to a new immune escape mechanism in NSCLC. In support of this view, we were able to detect infiltration of CD27^+^ lymphocytes in 90% of NSCLC specimens. Furthermore, tumor infiltrating lymphocytes surrounding CD70^+^ tumor cells, showed a trend towards increasing FOXP3 expression and higher CD4/CD8 ratios.

However, the expression of CD27 on regulatory T cells requires further examination to elucidate its role in anti-tumor immune responses in NSCLC. Nonetheless, it is already known that in glioblastoma, CD70 expression has been implicated in escape from immune surveillance [14].

It has previously been reported that activation of CD27 leads to the cleavage of sCD27 by metalloproteinases and shedding from the cell surface [18]. We are the first to reveal a potential role of soluble CD27 as a prognostic marker in NSCLC. High serum sCD27 levels were found to be significantly associated with poor OS and PFS and even though sCD27 levels were not predictive of CD70 overexpression on tumor cells, dual positivity of CD70 and sCD27 marked an even worse prognosis. The use of sCD27 as a prognostic marker is in accord with studies in hematological malignancies such as diffuse large B cell lymphoma and Waldenström macroglobulinemia [28-30]. However, further work is needed to verify the role of sCD27 as a prognostic biomarker in solid tumors such as NSCLC.

ARGX-110, a human CD70-specific monoclonal antibody which is currently undergoing Phase I clinical testing, is a strong antagonist of CD70/CD27 signaling and mediates destruction of CD70-expressing tumor cells through enhanced ADCC [21]. Our data show that a low dose of ARGX-110 (0.5μg/ml) induces efficient NK cell based lysis in CD70 expressing NSCLC cell lines, indicating the importance of CD70 expression patterns for an efficient ADCC activity. Finally, in this study, a positive correlation between IHC and flow cytometric analysis of CD70 expression was established, indicating that IHC is a suitable method for assessment of tumor samples for ADCC activity of ARGX-110.

In conclusion, we are the first to demonstrate the immunotherapeutic potential of targeting CD70 in NSCLC with a maximum NK-cell mediated cytotoxicity of ARGX-110 in CD70 expressing lung cancer cell lines. In contrast to the near absence of CD70 from normal tissues, analysis of paraffin-embedded biopsies of NSCLC revealed constitutive overexpression of CD70 in tumor cells. Moreover, this study has revealed a possible role of CD70/CD27 signaling in immune escape in NSCLC, since 90% of biopsies were infiltrated by CD27^+^ lymphocytes in the microenvironment of the tumor and showed a trend towards increased FOXP3 expression and higher CD4/CD8 ratios surrounding CD70^+^ tumor cells. Finally, serum sCD27, which can be easily measured in clinical practice, has shown potential as a prognostic marker for NSCLC.

## MATERIALS AND METHODS

### Patient selection and tissue specimens

Nineteen serum samples and 65 formalin fixed paraffin embedded (FFPE) specimens, for which the main characteristics are described in Table 1, were collected from 53 NSCLC patients. The average age (±SD) of the patients included in this study was 64 ± 10 (age range 38 to 82 years), The study was approved by the Ethics Committee of the Antwerp University Hospital and for serum sample collection, all patients signed an informed consent. Tissue specimens were fixed in 4% formaldehyde for 6-18 h and paraffin embedded on a routine basis. Matching serum samples for 19 surgical resection specimens, collected just before excision, were available. For 4 patients, core biopsies or fine-needle aspirates in cell blocks were used.

### Molecular analysis

For EGFR mutation analysis, DNA was extracted from FFPE tissue blocks using the QIAmp DNA FFPE tissue kit (Qiagen, Venlo, the Netherlands), according to the manufacturer's instructions. Initially EGFR mutations in exon 19, 20 and 21 were investigated using high-resolution melting analysis (HRMA), as described previously [31]. Standard sequencing was used to identify activating or inhibiting EGFR mutations. ALK translocations were identified by fluorescence in situ hybridisation (FISH) using the Vysis LSI ALK dual-colour, break-apart rearrangement probe in combination with the Vysis pre- and post-treatment kit IV (Abott Molecular, Des Plaines, IL, USA), according to the manufacturer's instructions [32].

### Immunohistochemistry (IHC)

Five *μ*m-thick sections were prepared from FFPE specimens. Sections were subjected to heat-induced antigen retrieval (HIER) by incubation in a high pH buffer for 20 min at 97°C (PT-Link)(DAKO, Glostrup, Denmark). Subsequently, endogenous peroxidase activity was quenched by incubating the slides in peroxidase blocking buffer (DAKO) for 5 min. Incubation with primary monoclonal antibodies anti-CD4 (Clone 4B12 ready to use for 20 min, DAKO), anti-CD8 (Clone C8/144b ready to use for 20 min, DAKO) and anti-CD70 (Clone 301731 diluted 1:40 for 20 min, R&D) was performed at room temperature on a DAKO autostainer Link 48 instrument using the Envision FLEX+ detection kit (DAKO) according to the instructions of the manufacturer. For CD70 expression, primary incubation was followed by incubation with mouse enhanced polymer-based linker (DAKO) for 30 min. Expression of CD27 (Clone 137B4 diluted 1:25 for 40 min, Thermo fisher scientific, Nepean, Canada) and FOXP3 (Clone 237A3/E7 diluted 1:200 for 40 min, Abcam, Cambridge, MA, USA) was assessed using the ultraview detection kit on a Ventana BenchMark ULTRA (Roche, Diagnostics GmbH, Mannheim, Germany) according to the manufacturer's instructions after HIER in a high pH buffer for 32 min at 95°C. Sections were counterstained with haematoxylin, dehydrated and mounted.

Positive controls were included in each staining run and consisted of tonsil tissue. Biopsies were checked for internal positive control. Scoring was performed by two independent observers as well as one pathologist, positive staining was assigned when at least 10% of the tumor cells, of any intensity (+, ++, +++) and any CD70 distribution (membranous, cytoplasmic) showed specific CD70 staining. In addition, the degree of staining was classified as follows: no staining (0), weak (+), moderate (++) and strong staining (+++).

### Cell lines and cell culture

The human NSCLC cell lines CRL-5908 and CRL-5883 were purchased from the American type cell culture collection (ATCC, Rockville MD, USA). The multiple myeloma cell line JJN-3, kindly provided by Prof. Dr. K. Vanderkerken (VUB, Brussels, Belgium), was used as a positive control because of its high percentage of CD70^+^ cells. JJN-3 cells were cultured in RPMI supplemented with 10% fetal bovine serum (FBS), 1% penicillin/streptomycin and 1% L-glutamine (Life Technologies, Merelbeke, Belgium). CRL-5908 and CRL-5883 were cultured in RPMI supplemented as described above with an addition of 1 mM sodium pyruvate (Life Technologies). Cells were grown as monolayers and maintained in exponential growth in a humidified 5% CO_2_/95% air atmosphere at 37°C. Cell cultures were confirmed free of mycoplasma infection through regular testing using the *MycoAlert® Mycoplasma detection kit (*Lonza, Verviers, Belgium).

### Determination of CD70 expression by flow cytometry

For flow cytometric analysis, 2.5 × 10^5^ cells were washed in buffer (1x phosphate buffered saline (PBS), 0.5% FBS), labelled with 100 μl ARGX-110 (50μg/ml) and incubated on ice for 30 min. Anti-human IgG1 PE antibody (diluted 1:200, eBioscience, San Diego, CA, USA), was applied as a secondary antibody in the dark for 30 min at 4°C. Thereafter, fluorescence was measured using a FACScan flow cytometer (BD, Heidelberg, Germany). As a negative control, cells were incubated with the secondary antibody without primary antibody.

### Purification and analysis of human NK cells

Peripheral blood mononuclear cells (PBMC) were isolated by Ficoll-Paque Plus gradient separation (Amersham Biosciences, Uppsala, Sweden) from buffy coat preparations of healthy donors, provided by the Antwerp Blood Transfusion Centre. CD56^+^CD3^−^ NK cells were obtained from PBMC using the Human Negative Selection NK Cell isolation kit (Miltenyl Biotec, Utrecht, The Netherlands) according to the manufacturer's instructions. NK cells were analysed on a Partec CyFlow ML cytometer (Partec, Münster, Germany), using FITC and PE-labelled monoclonal antibodies for CD56 and CD3 (BD Biosciences, Erembodegem, Belgium) whereby a purity of 85.3 ± 2.3 % (mean ± SD %, n=9) viable CD56^+^CD3^−^ NK cells were obtained. NK cells were resuspended in RPMI or DMEM, depending on further experiments.

### xCELLigence real-time cell analysis (RTCA): cytotoxicity

All experiments were carried out using the *xCELLigence* RTCA DP instrument (Roche Diagnostics GmbH, Mannheim, Germany), located in a humidified incubator at 37°C and 5% CO_2_. Cytotoxicity experiments were performed using modified 16-well plates (E-plate, Roche Diagnostics GmbH, Mannheim, Germany) [33]. Primarily, 100 μl of cell-free growth medium was added to the wells. After 30 min of incubation at room temperature, the background impedance for each well was measured. Thereafter, cells were harvested by a standardized detachment procedure using 0.05% Trypsin-EDTA (Life Technologies, Merelbeke, Belgium) and counted automatically with a Scepter 2.0 device (Merck Millipore SA/NV, Overijse, Belgium). Fifty μl of the cell suspension, containing 10,000 cells, was seeded into the wells, left at room temperature for 30 min and locked in the RTCA DP device. From then on the impedance value of each well was automatically monitored every 15 min by the xCELLigence system and expressed as a cell index value (CI). In each test, two replicates of the same conditions were used and run in parallel with NK cells of three different donors. Twenty-four hours after cell seeding, 10 μl of ARGX-110 (0.5 μg/ml, final concentration) diluted in PBS was added to each well. Human immunoglobulin G1 (IgG1), lambda purified from myeloma plasma (Sigma, I5029), was used as an isotype control. 20 μl cell-free medium or effector CD56^+^CD3^−^NK cells were then added to each well at E:T ratios of 0:1, 1:1, 5:1 or 10:1. Three days after the start of treatment with ARGX-110, CI measurement was ended.

### sCD27 levels: ELISA

Serum soluble CD27 levels were measured using the PeliKine Enzyme-Linked Immunosorbent Assay (ELISA) kit (Sanquin Reagents, Amsterdam, the Netherlands). Assays were carried out according to the manufacturer's instructions. Briefly, serum sCD27 was measured after binding to a monoclonal CD27 antibody coated to a polystyrene microtiter plate. After rinsing, a biotinylated second monoclonal CD27 antibody was added. After 30 min incubation, excess biotinylated antibody was removed by washing, followed by addition of horseradish peroxidase (HRP) conjugated streptavidin. After 30 min of incubation, non-bound streptavidin-HRP conjugate was removed by washing and substrate solution was added. After termination of the reaction by the addition of a stop solution, absorbance was measured at 450 nm (Biorad Microplate reader, Temse, Belgium).

### Statistical analysis

Prognostic relevance of *sCD27, CD27 and CD70* was assessed by survival analysis. The date of diagnostic confirmation for NSCLC was used as the index date for survival time calculation. Overall survival was calculated from the index date to the date of last information/death. For progression-free survival, the months of observation were calculated from the index date to the first date of progression or the date of last information. The Kaplan-Meier method was used for estimation of survival probability. Differences were analyzed with the log-rank test. Furthermore, possible associations with clinicopathological parameters of NSCLC were examined using the *χ*^2^-test or Fisher's exact test (when appropriate) for categorical variables and using Student *t*-test or Mann–Whitney *U*-test (when appropriate) for continuous variables. In addition, statistical significance for *in vitro* experiments was determined by a one-way ANOVA test, followed by Tukey's post hoc test. All analyses were conducted using SPSS version 22 (SPSS Inc., Brussels, Belgium) Significance for all statistics was reached if *P* < 0.05 (two tailed).

## SUPPLEMENTARY FIGURE


