# Supplementary material for: Unlocking the potential of CD70 as a novel immunotherapeutic target for non-small cell lung cancer
Source: Oncotarget. 2015 Apr 19;6(15):13462–75. doi: 10.18632/oncotarget.3880 (PMC4537027; doi:10.18632/oncotarget.3880)
Supplement: Supplementary file 1 [file oncotarget-06-13462-s001.pdf]

Unlocking the potential of CD70 as a novel immunotherapeutic target for non-small cell lung cancer

Supplementary Material

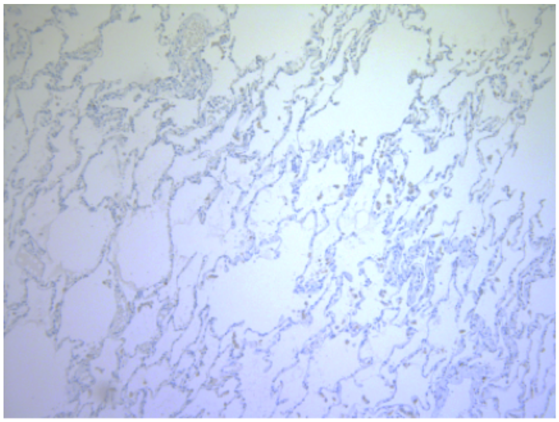

Supplementary figure 1: CD70 staining in normal lung tissue.
